# Prevention of catheter-associated urinary tract infection: implementation strategies of international guidelines^1^

**DOI:** 10.1590/1518-8345.0963.2678

**Published:** 2016-03-28

**Authors:** Vera Lúcia Fonseca Andrade, Filipa Alexandra Veludo Fernandes

**Affiliations:** 2 MSc, Assistant Professor, Escola Superior Politécnica de Saúde, Instituto de Ciências da Saúde, Universidade Católica Portuguesa, Lisboa, Portugal; 3 MSc, RN, Unidade de Cuidados Intensivos, Centro Hospitalar Barreiro Montijo, EPE, Barreiro, Portugal

**Keywords:** Urinary Tract Infections, Urinary Catheterization, Nursing

## Abstract

**Objective:**

to describe strategies used by health professionals on the implementation of the Centers for Disease Control and Prevention guidelines for the prevention of urinary infection related to catheterism.

**Method:**

systematic review on literature based on data from CINAHL(r), Nursing & Allied Health Collection, Cochrane Plus Collection, MedicLatina, MEDLINE(r), Academic Search Complete, ACS - American Chemical Society, Health Reference Center Academic, Nursing Reference Center, ScienceDirect Journals and Wiley Online Library. A sample of 13 articles was selected.

**Results:**

studies have highlighted the decrease of urinary tract infection related to catheterism through reminder systems to decrease of people submitted to urinary catheterism, audits about nursing professionals practice and bundles expansion.

**Conclusion:**

the present review systemizes the knowledge of used strategies by health professionals on introduction to international recommendations, describing a rate decrease of such infection in clinical practice.

## Introduction

Catheter-associated urinary tract infection (CAUTI) is frequent on hospitalized people. On infections associated to health care, catheter-associated urinary tract infection corresponds to 40%^(^ 1 ^)^_._ About 15% to 25% of hospitalized people are submitted to vesical catheterization^(^ 2 ^)^. In most cases, this technique is used without proper indication, extending its unnecessary use. CAUTI is referred to in several studies about increase on mortality and morbidity, as well as hospitalization, and, consequently, associated costs^(^ 2 ^)^.

However, it is pointed out that when compared to other healthcare-associated infections (HAI), urinary tract infection (UTI) presents a low morbidity. A study carried out in the USA^(^ 2 ^)^ determined that UTI rate was superior to 560 thousands when compared to other HAIs, with a mortality rate of 2.3%, and bacteriuria (which develop bactareamia) cases lower than 5%. UTI is the main cause of infecctions on secondary nosocomial bloodstream, about 17% of nosocomial bactareamia origin from urinary, with an associated mortality of 10%. Society for Healthcare Epidemology of America (SHEA) estimates between 17% and 69% of CAUTI can be prevented through infection control recommendations, based on evidences^(^ 2 ^)^_._

The investigation suggests that CAUTI prevention goes through recommended actions, and based on evidences that lower the rate for this infection. Simple initiatives as hand hygiene, use of a reliable technique, maintenance and the way the catheter is removed can contribute to prevent the associated infection. An unnecessary urinary catheterism and the period of permanency of catheter influence the infection development, being modifiable factors^(^ 2 ^)^. Currently, the practice based on evidences is a highlight in a culture that aims a quality standard for safe care. However, it is pointed out that many times professionals face a few embarrassments regarding the connection between results from the most current and practical investigation. The way actions and/or strategies are applied for implementations, in one document, providing practical changeable tools to readers, based in measurable results. This systematic review supplies the answer to this need, gathering the scattered knowledge on literature and facilitating the access to safe practices based on evidences. The study's goal was to search evidences regarding strategies that health professionals found out on practice to apply/implement of CDC recommendations and CAUTI prevention. 

## Method

The structure of this systematic review on literature was based on the CAUTI thematic, elaborating the initial question, defining eligibility criteria, selecting article samples and further analysis and results discussion. To obtain the answer to the initial question "What are the strategies health professionals use for implementing the Centers for Disease Control and prevention (CDC) guidelines for CAUTI prevention?". A systematic review of the literature was made to reach this goal. 

As a research strategy, the following health descriptors were used: urinary AND infection AND catheter AND nurs*. To find answers to this question, the goal was to perceive how nurses operationalized the recommendations of the Centers for Disease Control and prevention on praxis and intervention on CAUTI prevention.

**Figure 1:**
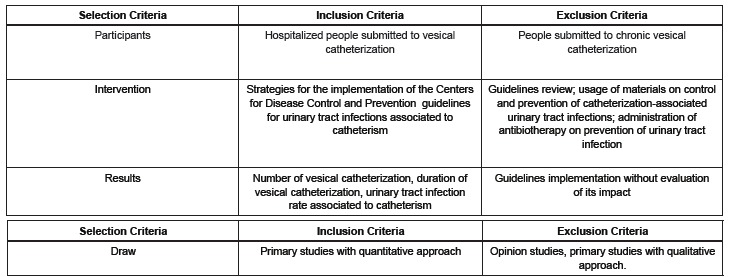
- Inclusion and exclusion criteria

The temporal limit of research was from January 2007 to December 2014, for fully available papers, as a way to contextualize the thematic in the past seven years. The databases used were CINAHL^(r)^, Nursing & Allied Health Collection, Cochrane Plus Collection, MedicLatina, MEDLINE^(r)^, Academic Search Complete, ACS - American Chemical Society, Health Reference Center Academic, Nursing Reference Center, ScienceDirect Journals, and Wiley Online Library. Face to the studied phenomenon, the eligibility criteria were established according to Figure 1.

**Figure 2:**
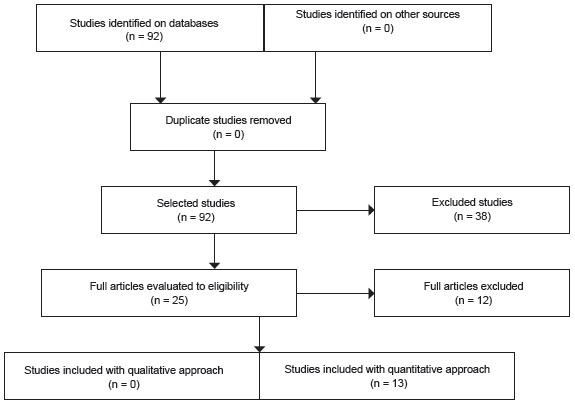
- Flowchart of articles selection

We would like to highlight that the exclusion of articles was also related to lack of information about parameters considered important for the analysis; participants, interventions, results, study's draw. Facing the studies' diversity and non-relevance to the investigation question, the parameter*Comparison* [C] was removed. We also considered, when selecting the studies, the bibliographic references mentioned^(^ 3 ^)^_._ On the initial phase, 92 articles were obtained, from which, 38 were excluded by their titles. After reading 53 abstracts, 29 articles were rejected. Twenty-five articles were fully analyzed, from which 12 were rejected, according the inclusion/exclusion criteria, leading to 13 articles selected to perform the systematic review on literature. Figure 2 is a flowchart of the mentioned article selection. 

**Figure 3:**
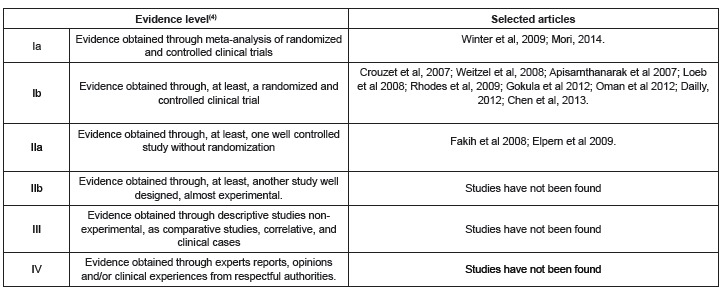
- Rating of selected articles, according to evidence level

Afterwards, the articles were classified in accordance to evidence level^(^ 4 ^)^, determining that most of the selected articles presented a high level of evidence, considering about 70% are on evidence level Ib. On evidence level Ib, the scientific evidence comes from, at least, a random clinical trial. Following two articles placed on evidence level IIa, and two articles on level Ia, the last one being the higher evidence level^(^ 4 ^)^. On level IIa, the evidence was obtained through a forward study controlled and well designed, without randomization. On level Ia, the scientific evidence obtains through a met-analysis of random clinical trials. The 13 selected articles, according the evidence level, are presented on Figure 3.

## Results

**Figure 4:**
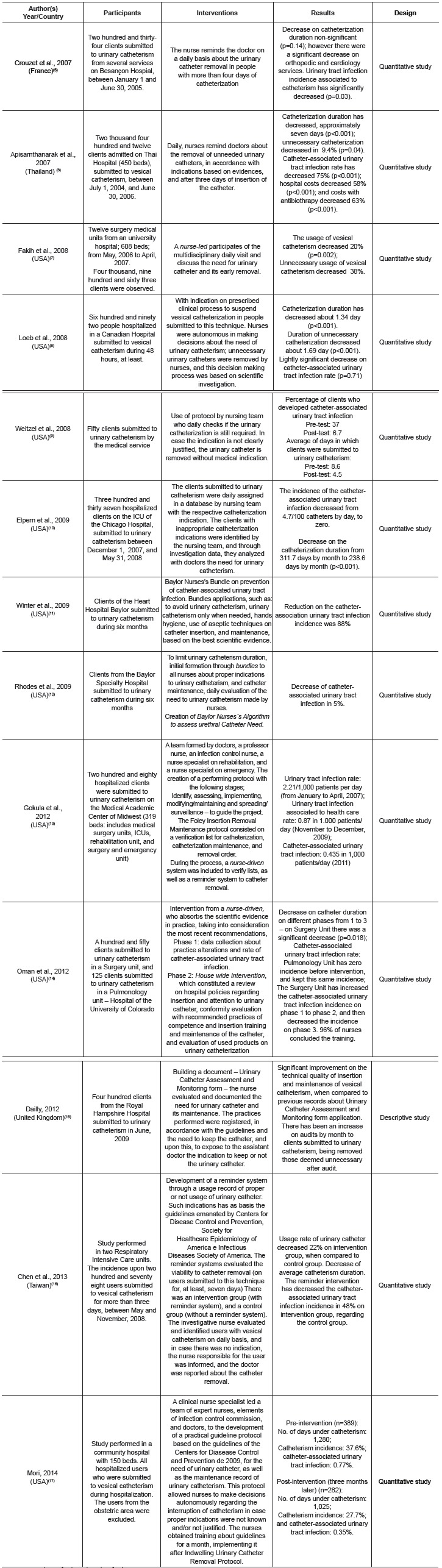
- Analysis of selected studies for literature review

The results are presented in table, with the analysis of studies selected to review literature, specifying each one according to author, year, country, participants, interventions, results and design (Figure 4). We made a detailed analysis of these factors regarding their contribution to the answer to the investigation question. 

## Discussion

In the search for better evidence, we concluded that the articles present a high level of evidence for the answer to the matter being investigated. They are in between the levels I and II of evidence^(^ 4 ^)^, promoting a more consistent validity for the resolution of the problem.

The number of participants in the analyzed studies varied between 50 and 4,963. In two retrospective studies ^(11-12)^ what was defined was the time of development of the studies, and not the number of clients.

Although big difference between the number of participants in different studies, having groups with more than 2,000^(^ 6 ^-^ 7 ^)^, the results were similar in comparison to smaller groups. We determined that, despite the difference in the methodology applied, the results of the studies are analogous. In the majority of them, there was an intervention phase and a post-intervention one^(^ 6 ^-^ 7 ^,^ 9 ^-^ 10 ^,^ 13 ^-^ 14 ^,^ 17 ^)^. Some studies motivate the formation of nurses about the guidelines of prevention of CAUTI, revealing extreme importance in their duties ^(^ 9 ^,^ 11 ^-^ 15 ^,^ 17 ^)^.

Taking into account the obtained results, according to Figure 4, they all respond to the matter of the investigation: "What are the strategies health professionals use for implementing the Centers for Disease Control and prevention (CDC) guidelines for CAUTI prevention?", once they demonstrate the intervention of the health professionals in the implementation of different strategies for the response to the matter of CAUTI and obtaining effective results.

By making the audit document^(15)^ - UCAM - based on scientific recommendation for the prevention of CAUTI, the nurses managed to improve the records under the manipulation done in the technique of vesical catheterization. The records, as indicators of vesical catheterization and evaluation of the necessity to keep the catheter, have motivated the removal of the catheters considered unnecessary ^(^ 15 ^-^ 17 ^)^. According to the guidelines of CDC ^(^ 2 ^)^, the early removal of the urinary catheter contributes for the prevention of the infection associated to this device.

One of the strategies used by the nurses was to remind the doctor about the necessity of the urinary catheter^(^ 5 ^-^ 6 ^,^ 10 ^)^. The justification used by the nurses was based on appropriate indicators described on scientific evidence, concerning the need of the urinary catheter. In the cases in which the catheters were not removed, the duration of the catheterization decreased, so did the incidence of CAUTI.

The dominant strategy throughout the studies is the application of bundles of many formats in the prevention of CAUTI ^(^ 7 ^-^ 9 ^,^ 11 ^-^ 14 ^)^. The term bundle was developed by the Institute for Healthcare Improvement ^(18)^ to describe a set of intervention, based on evidence and directed to the clients/population who are under inherent risks care. This set of intervention, when implemented together, originate significantly better results than those implemented individually^(^ 18 ^)^. The bundle used were related to the insertion technique and the maintenance of the vesical catheter, to avoid the urinary catheter, and also, limiting its duration.

Another identified strategy was the creation of the algorithm for the maintenance of the urinary catheter, based on evidence, whenever really necessary ^(^ 12 ^,^ 17 ^)^. On the other hand, when the indication for the usage of catheter was not present, it was removed after analysis with the doctor. With the application of the algorithm it was subjacent the daily evaluation of the necessity of the urinary catheter. Both studies allowed the reduction of the usage of the vesical catheter and the incidence of CAUTI.

With the purpose of assessing the necessity of the urinary catheter on clients, the impact of the daily participation of a nurse with management duties (nurse-led) was evaluated in the multidisciplinary visit and the assessment proposing the removal of the catheter ^(^ 7 ^)^. In this study, there is a decline of the usage and unnecessary usage of the vesical catheter. The decision making is based on evidence of the nurses in suspending the vesical catheterization. The nurses were based on the recommendation of scientific evidence for the making of protocols of performance in the prevention of CAUTI, from its insertion and maintenance, until the evaluation of the necessity of catheterization^(9,13-14,16-17).^

In summary, the health professionals, based on CAUTI prevention guidelines, present many methods of implementation, responding, in an efficient way, to this problematic. The attention during the procedure, handling of the vesical catheter, duration of the catheterization and staff training are of extreme importance^(19-20)^. Determining the crucial and decisive role that the health professional play in the prevention and control of urinary infection associated to urinary catheter. The awareness of the professionals for this problematic is a fundamental starting point for this supported practice, seen in some studies^(^ 9 ^,^ 11 ^-^ 15 ^,^ 17 ^)^ in which education was one of the tools for the starting point of this change.

 The strategies applied in the implementation of guidelines have lessened the incidence of CAUTI, however, they appear in the literature in a disperse way. This review brings up knowledge, once it systematizes results of the practice and it allows the health professionals access to effective, up-to-date infection control strategies, with positive results in the quality of health care.

As limitations of this study, we highlight the process of selecting the sample done only by a reviewer and the usage of the nurs* descriptor on the research strategy. The critic analysis of the criteria on the series of studies by two reviewers would result better consistency in this review^(^ 21 ^)^, as well as, the absence of the nurs* descriptor associated to the research to other professionals and, consequently, other strategies of practical implementation of international recommendation. At last, the selected sample for the review was of a reduced number and more studies are necessary to increase the number of strategic evidence of implementation, as well as its contribution for effective changes in care practice.

## Conclusion

 The intervention based on evidence can promote the evaluation of the necessity of urinary catheterization and removing the catheter when not necessary and allowing the reduction of the CAUTI rate.

In relation to the goal of this study and in summary, the main strategies of implementation of the guidelines were the performance of audition to the procedure, reminder systems for the assessment of the necessity of urinary catheterization and the implementation of bundles. The strategies found by the nurses to respond to this problematic were efficient, with the development of the health team, based on the prevention and control of the infection and the improvement of the safety of the person who seeks the health services and are put through this technique. In practice, policies should be implemented to promote the early removal of the catheters, as well as, those that are not necessary, avoiding negative consequences to the client and the institution, namely the hospitalization period. This review, besides systematizing the strategies found by the health professional in the implementation of the guidelines in the prevention of CAUTI, opens the gates to new investigation on this area, as a starting point and gaps that can arise. It is important to emphasize the necessity of more studies about the intervention of health professionals in the performance and maintenance of the vesical catheterization technique and, consequently, in the prevention of CAUTI. The strategies described by them are not enough, they need health professionals awareness and motivation on this problematic and studies on other sections that can contribute to the prevention and control of infection associated to health care.
